# Three-Dimensional Computational Model Simulating the Initial Callus Growth during Fracture Healing in Long Bones: Application to Different Fracture Types

**DOI:** 10.3390/bioengineering10020190

**Published:** 2023-02-02

**Authors:** José M. Naveiro, Luis Gracia, Jorge Roces, Jorge Albareda, Sergio Puértolas

**Affiliations:** 1 Department of Mechanical Engineering, University of Zaragoza, 50018 Zaragoza, Spain; 2 Aragón Institute for Engineering Research, 50018 Zaragoza, Spain; 3 Department of Construction and Manufacturing Engineering, University of Oviedo, 33204 Gijón, Spain; 4 Department of Surgery, University of Zaragoza, 50009 Zaragoza, Spain; 5 Aragón Health Research Institute, 50009 Zaragoza, Spain

**Keywords:** diaphyseal fractures, bone callus formation, bone growth factors, finite element analysis, automatic mesh generation

## Abstract

Bone fractures are among the most common and potentially serious injuries to the skeleton, femoral shaft fractures being especially severe. Thanks to recent advances in the area of in silico analysis, several approximations of the bone healing process have been achieved. In this context, the objective of this work was to simulate the initial phase of callus formation in long bones, without a pre-meshed domain in the 3D space. A finite element approach was computationally implemented to obtain the values of the cell concentrations along the whole domain and evaluate the areas where the biological quantities reached the thresholds necessary to trigger callus growth. A voxel model was used to obtain the 3D domain of the bone fragments and callus. A mesh growth algorithm controlled the addition of new elements to the domain at each step of the iterative procedure until complete callus formation. The implemented approach is able to reproduce the generation of the primary callus, which corresponds to the initial phase of fracture healing, independently of the fracture type and complexity, even in the case of several bone fragments. The proposed approach can be applied to the most complex bone fractures such as oblique, severely comminuted or spiral-type fractures, whose simulation remains hardly possible by means of the different existing approaches available to date.

## 1. Introduction

Bone fractures are one of the main issues in healthcare, femoral shaft fractures being among the most severe injuries of the skeleton. Although these currently account for only 0.9% of fractures [1], when they occur, they are associated with high morbidity and mortality [2,3]. Although they can occur in young patients due to high-energy trauma, currently 85% of patients are elderly, mainly women with osteoporotic bone [4]. When a femur fracture occurs, a common surgical procedure is to implant an intramedullary nail (IM). This surgery is not easy and has some risks including infection, morbidity and pain [5].

After the surgical procedure, the bone recovery goes through various phases that overlap. These phases can be classified as: acute inflammatory response, recruitment of mesenchymal stem cells, generation of a cartilaginous and a periosteal bony callus, revascularisation and neoangiogenesis at the fracture site, mineralisation and resorption of the cartilaginous callus, and bone remodelling [6].

Several studies concerning the clinical assessment of callus formation by means of image or ultrasound techniques have been published during recent years [7,8,9,10,11], but they only provide partial information attached to specific steps without the complete evolution; moreover, the results correspond to specific fractures and patients, and it is hard to extrapolate those findings to other different conditions.

On the other hand, experimental testing in animal models has been used for the study of the early period of fracture healing [12,13,14], but these models can hardly be applied to humans, due to the differences at the anatomical level and between in vivo and in vitro behaviour.

Those difficulties have led to the development of simulation models using the finite element method (FEM), allowing the assessment of the influence of the different magnitudes involved in the problem, and facilitating the reproduction of the considered studies under different conditions.

In the field of computational models applied to bone fracture analysis, great improvements have been made in the last two decades. Some works are focused on analysing the influence of different factors in tissue fracture healing [15,16,17,18,19,20,21]. Other works are related to tissue differentiation through the implementation of fuzzy logic [22,23,24,25,26]. In other works [18,27,28], the migration of the necessary cells for osteosynthesis is simulated by the diffusion problem. 

There are a few works with a pre-defined mesh approach. Lipphaus et al. [29] simulated femoral shaft fractures with initial interfragmentary translation movement. The simulation was based on a linear elastic material model and included the generation of fracture haematoma and initial mesenchymal stem cell concentration out of an unspecified solid, cell proliferation, migration, and differentiation due to mechanical stimuli and time-dependent axial loading. Wang et al. [25] made 3D simulations regulated with fuzzy logic rules, and they included the simulation of tissue differentiation. An ovine tibia was modelled based on the animal models in the work of Claes et al. [30]. Finally, Syed Hasan Askari Rizvi et al. [21] analysed the effects of a composite IM on cell-phenotype-related activities and callus growth during the healing of tibial bone fractures. To do this, they appended a mechanoregulation algorithm. They modelled a healthy male tibia as a 300 mm cylinder and then reproduced a 3 mm gap transverse fracture at the mid-diaphyseal shaft. 

All of the published works concerning this subject have a pre-meshed domain where the fracture callus growth takes place. A pre-meshed domain is a domain that has previously been meshed assuming the callus shape. No works with a free mesh approach could be found in the literature. However, in a previous work a new approach was presented by the authors [31] by which callus development proceeded without a pre-defined meshed domain that restricted and directed callus growth. In that work, the proposed approach was applied to axisymmetric models of femoral fractures treated by means of intramedullary nailing, with reliable results.

The main goal of the present work was to apply the approach published in a previous work [31] to a general three-dimensional formulation, in order to be applied to femoral fractures with different patterns. Several computational models, based on real cases obtained by magnetic resonance imaging (MRI), were used for different types of femoral fractures, allowing the validation of the extended formulation. The potential clinical value of this approach lies in helping orthopaedic surgeons to predict the times required for fracture callus formation depending on the type and complexity of the fracture. Moreover, the personalized simulation of specific patients and fractures could be performed, for example from computed tomography (CT) images.

## 2. Materials and Methods

The present approach is intended only for callus growth, focusing on the simulation of the initial stage of callus formation. 

The problem was approached through a diffusion process that emulates the movement of cells through the bone up to the fracture zone, triggering the callus growth when a certain threshold level is reached. To solve the diffusion process, a finite element model was implemented considering two different types of cells. Furthermore, the free growth meshing algorithm is an automatic mesh generator that allows working without a pre-meshed domain, generating the mesh inasmuch as the growth occurs. To avoid pre-meshing, there was a free growth space on the outside of the bone geometry so as to obtain the initial callus shape with the free growth meshing algorithm. The combination of the finite element model and the free growth meshing algorithm resulted in a global algorithm that was iterated over time until progress was made in the formation and closure of the fracture. This iterative algorithm allows knowing the state of the bone mesh and callus mesh at each step. In order to simulate the diffusion problem, it is necessary to choose the most relevant cellular factors. For this reason, mesenchymal stem cells (MSCs) and chondrocytes were chosen. There are many types of cells involved in bone healing. MSCs and chondrocytes were considered the most important types of cells in the generation of this initial callus [16] because they are the main precursors of its formation. They were therefore considered in the diffusion model including and evaluating their concentrations.

In addition to cellular factors, it is necessary to consider the influence of chemical factors that condition the rate of fracture callus formation. In this study, tumour necrosis factor α (TNF-α) and the bone morphogenetic protein 2 (BMP-2) were considered. The TNF-α molecule plays a major role in the inflammatory stage to regulate the immune system, prior to callus formation [32]. However, this molecule is also present in the phases that require the recruitment of MSCs to perform its most important mission: to control the apoptosis of chondrocytes, which will leave their calcified extracellular matrix. This process will initiate the reabsorption of calcified cartilage during later stages [6]. Regarding BMP-2, these molecules belong to the transforming growth factor β (TGF-β) superfamily [6]. This superfamily has been widely analysed in clinical applications [6,32]. In particular, BMPs are molecules that accelerate the process of bone generation, helping MSCs in the process of cell differentiation into osteoblasts and chondrocytes, and accelerating the union time [33].

Furthermore, it is possible to add new factors if necessary and incorporate them into the proposed algorithm.

The proposed approach consists of three phases:

1. Cells diffusion problem.

This phase corresponds to the diffusion problem concerning biological magnitudes that control callus formation (MSCs and chondrocytes). The problem is numerically solved by means of the implementation of an appropriate finite element analysis (FEA) formulation.

2. Checking when the biological magnitudes reach the growth threshold.

Once the cell concentrations are calculated, the second phase identifies the elements that have free faces at the fracture edge and which concentrations are above the callus growth trigger threshold. These elements have a growth velocity that can be influenced by the presence of drugs and other possible chemical parameters, according to a sigmoid function.

3. Callus mesh growth algorithm.

In this phase, the mesh geometry is updated by adding new elements on the fracture edge, following the previously calculated directions. For each time iteration, the mesh growth algorithm generates new elements developed over the previously existing elements. This phase closes each simulation step.

**Figure 1:**
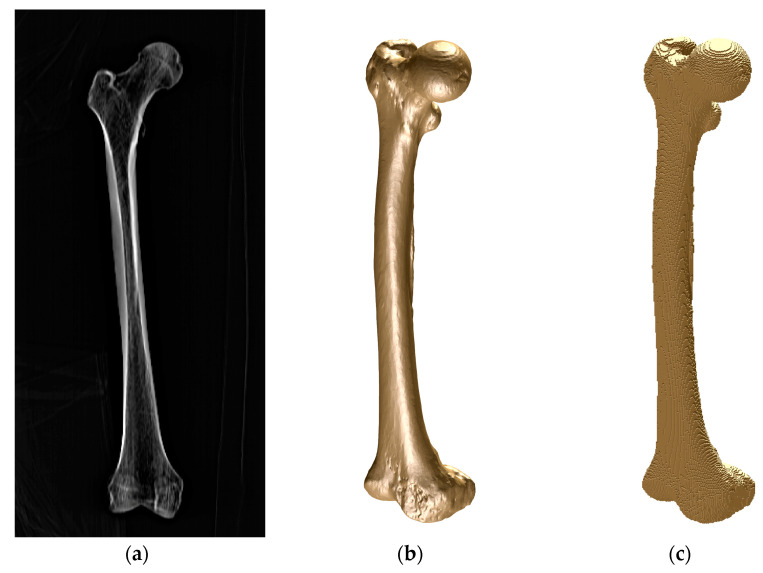
Femur of a 46-year-old man: (**a**) frontal MRI image; (**b**) CAD geometrical model; (**c**) voxel model.

The geometrical model of the femur was obtained from a real femur of a 46-year-old male (Figure 1) by means of MRI. Each image is a layer in which the bone section appears in a grey scale. The distance between layers was 1 mm in the direction of the natural axis of the femur. The tissue density in relation to the Hounsfield units was calculated from the images. Once the images were processed, the MRI was imported to the modVOX^®^ program to obtain the voxel model [34]. The voxel model was composed of 1 mm cubes.

**Figure 2:**
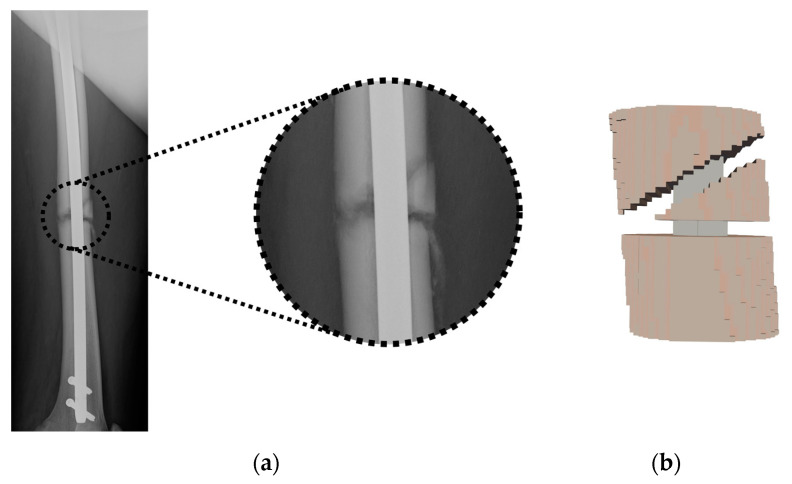
Selected region from the femur fracture: (**a**) radiological image; (**b**) voxel model.

Only a fraction of the complete voxel model was considered for the simulations, corresponding to the zone of interest around the fracture site (as shown, for example, for a comminuted fracture in Figure 2). Three different materials were considered: nail, trabecular bone (which also includes the periosteum) and cortical bone.

### 2.1. Diffusion Problem: Numerical Approach

MSC and chondrocyte cell concentrations at the fracture focus are responsible for the activation of callus growth, while the TNF-α and BMP-2 factors regulate the velocity of the fracture healing. Therefore, a diffusion problem was formulated for each of these two magnitudes in order to calculate the corresponding concentrations from the initial conditions. Fick’s second law was used to address how diffusion causes the cells’ concentrations to change with respect to time.

Fick’s second law is expressed by the following differential equation:

(1) $$\frac{dc}{dt}=\nabla \left(D\nabla c\right)$$

where *c* is the cells’ concentration, *D* is the diffusion coefficient and *t* is the time; ∇ is the divergence/gradient operator. According to the formulation of the diffusion problem, the parameter *D* physically corresponds to the dispersion relation of a particle in a specific medium, in such a way that an increase in this parameter stimulates the propagation of the particles in the medium and a decrease in this parameter produces the opposite effect. According to the literature [16,17,27], the parameter was considered constant in every region. Then, fixing *D* as a constant value, the above equation can be written as follows:

(2) $$\frac{dc}{dt}=D\Delta c$$

with Δ being the Laplacian operator. For the finite element approach of the differential equation, the usual weak formulation and its subsequent discretization were applied. Thus, the initial equation can be written in the form:

(3) $$\overset{Ne}{\underset{e=1}{\sum }}\overset{Nne}{\underset{i=1}{\sum }}\overset{Nne}{\underset{j=1}{\sum }}\left[\frac{dc_{i}^{e}}{dt}\overset{}{\underset{\Omega^{e}}{\int }}\;\varphi_{i}^{e}\varphi_{j}^{e}\;d\Omega^{e}\right]\;\;=\overset{Ne}{\underset{e=1}{\sum }}\overset{Nne}{\underset{i=1}{\sum }}\overset{Nne}{\underset{j=1}{\sum }}Dc_{i}^{e}\overset{}{\underset{\Gamma^{e}}{\int }}\nabla \varphi_{i}^{e}\varphi_{j}^{e}\;d\Gamma^{e}-\overset{Ne}{\underset{e=1}{\sum }}\overset{Nne}{\underset{i=1}{\sum }}\overset{Nne}{\underset{j=1}{\sum }}Dc_{i}^{e}\overset{}{\underset{\Omega^{e}}{\int }}\;\;\nabla \varphi_{i}^{e}\;\nabla \varphi_{j}^{e}\;d\Omega^{e}\;$$

where $c_{i}^{e}$ is the nodal value of concentration for each element (depending on time), $\varphi_{i}^{e},\;\varphi_{j}^{e}$ are the corresponding elemental approximation functions, *Ne* is the number of elements in the mesh, *Nne* is the number of nodes of the element, $\Omega_{e}$ the elemental domain and $\Gamma_{e}$ represents the elemental boundary. The type of elements used were hexahedra with a linear approximation. Using the above formulation, the following elemental matrices and vector can be obtained:

(4) $$M^{e}=\overset{Nne}{\underset{i=1}{\sum }}\overset{Nne}{\underset{j=1}{\sum }}\overset{}{\underset{\Omega^{e}}{\int }}\;\varphi_{i}^{e}\;\varphi_{j}^{e}\;d\Omega^{e}\;$$

(5) $$N^{e}=\overset{Nne}{\underset{i=1}{\sum }}\overset{Nne}{\underset{j=1}{\sum }}D\overset{}{\underset{\Omega^{e}}{\int }}\;\nabla \varphi_{i}^{e}\;\nabla \varphi_{j}^{e}\;d\Omega^{e}\;$$

(6) $$f^{e}=\overset{Nne}{\underset{i=1}{\sum }}\overset{Nne}{\underset{j=1}{\sum }}D\overset{}{\underset{\Gamma^{e}}{\int }}\nabla \varphi_{i}^{e}\;\varphi_{j}^{e}\;d\Gamma^{e}\;$$

These elemental matrices were assembled, obtaining the following system of differential equations in time derivative:

(7) $$M\frac{dc}{dt}=f-Nc$$

Then, a finite difference scheme was used in order to solve the evolutionary problem defined by Equation (7). The following time discretization was applied to solve it:

(8) $$c_{t+\Delta t}=c_{t}+\Delta t\;\frac{dc_{t}}{dt}$$

Therefore:

(9) $$\frac{dc_{t}}{dt}=\frac{c_{t+\Delta t}-c_{t}}{\Delta t}$$

and the iterative algorithm was established as:

(10) $$\left(\frac{1}{\Delta t}M+N\right)\;c_{t+\Delta t}=f+\frac{1}{\Delta t}\;M\;c_{t}$$

**Figure 3:**
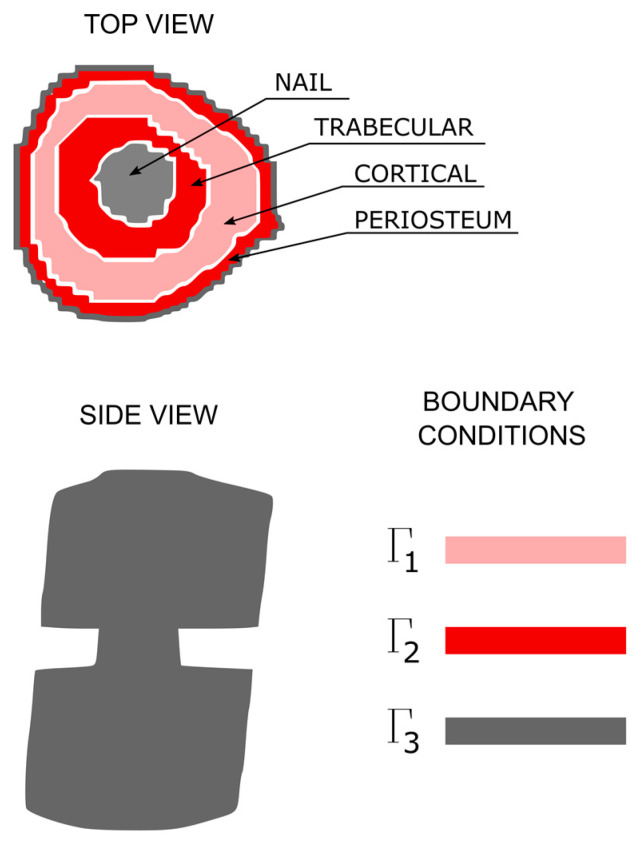
Boundary conditions in the 3D model representing a diaphyseal section of the femur with a transverse fracture.

The algorithm defined by Equation (10) was applied to the MSC and chondrocyte concentrations, with the following definition of the boundary conditions:

(11) $$\Gamma_{}=\Gamma_{1}\cup^{\text{​}}\Gamma_{2}\cup^{\text{​}}\Gamma_{3}\left\{\begin{array}{c} c\left(\vec{r},t\right)=D_{1}\;\Gamma_{1}\left(Essential\;conditions\right)\\ c\left(\vec{r},t\right)=D_{2}\;\Gamma_{2}\left(Essential\;conditions\right)\\ D\;\nabla c\;\vec{u}=0\;\;\Gamma_{3}\;\;\;\;\;\;\;\left(Natural\;conditions\right) \end{array}\right\}$$

with *Γ* being the whole boundary, where *Γ*_1_ and *Γ*_2_ represent boundary conditions 1 and 2 in which the concentration is prescribed, and *Γ*_3_ represents boundary condition 3 with zero flow rate (Figure 3). There were three different materials with three different diffusion coefficients: zero diffusion (the nail), low diffusion (the cortical region) and high diffusion (the trabecular and periosteal regions). 

The boundary *Γ*_2_ applies to the trabecular and periosteal region and is coloured in red. These regions are supposed to have a greater concentration of cells than the low-diffusion region corresponding to cortical bone. The nail has no concentration at all and is a zero-diffusion region. The low-diffusion region has a lower concentration than the red-coloured region and applies to the boundary condition *Γ*_1_; its colour is pink. It is supposed that no flux of cells passes from inside the bone to the exterior; this absence of flux is represented by the dark grey region representing the boundary condition *Γ*_3_. Figure 3 shows the differentiated parts of the bone and the boundary conditions for each part.

A specific program was developed and implemented in the FORTRAN language [35] to solve this finite element model.

### 2.2. Callus Growth Trigger and Control

This phase analyses which elements can grow and in which direction. The most commonly used method to detect the free faces is to create the inverse connectivity matrix of the nodes and detect which ones have less than five connected elements. However, due to the order of the voxel model, another method was implemented. The algorithm calculates the centroids of the elements. The centroids are used in the step of the callus mesh growth algorithm, so to take advantage of these data, the algorithm looks for the nearby centroids. If there is no centroid in some direction, then this means that in the chosen direction there is a free face and therefore it is susceptible to growth.

After detecting the elements with free faces, the cell concentrations at the nodes of the element are compared with the respective trigger thresholds. If the concentrations are below the threshold value, the corresponding face is not considered as a candidate for growing. For the growing faces, the cell concentration values determine the growth velocity through a sigmoid-like function (the same sigmoid-like function described in [31], appropriate for various biological processes [36,37,38]):

(12) $$v=v_{max}\;\varphi \left(\alpha ,\beta \right)$$

(13) $$\varphi \left(\alpha ,\beta \right)=\frac{4A}{\left[1+e^{-a\left(\alpha -\alpha_{0}\right)}\right]\left[1+e^{-b\left(\beta -\beta_{0}\right)}\right]}+B$$

where $v_{max}$ represents the maximum growth velocity, and its value is set according to known physiological values or indirectly through the usual fracture healing times observed in clinical practice [6,39].

The variables (*α*, *β*) are the dimensionless concentrations of MSCs and chondrocytes (*α* = c_MSCs_/c_MSCs_^máx^, *β* = c_cho_/c_cho_^máx^). *φ(α*,*β)* is a sigmoid-like normalized parametric function. It is defined in the [*α_0_*,1] × [*β_0_*,1] domain with *(α*_0_, *β*_0_*)* as the dimensionless thresholds of each concentration. The parameters *a* and *b* are positive constants that adjust the slopes of the function and are related to the concentrations of the chemical factors (TNF-α and BMP-2) involved in the rate of callus growth. The constants *A* and *B* are used to normalize the function and are defined as follows:

(14) $$\varphi \left(\alpha_{0},\beta_{0}\right)=0\;\;\varphi \left(1,1\right)=1\;$$

(15) $$A=\frac{k}{4-k}\;B=-A$$

With:

(16) $$k=\frac{1}{\left[1+e^{-a\left(1-\alpha_{0}\right)}\right]\left[1+e^{-b\left(1-\beta_{0}\right)}\right]}\;$$

**Figure 4:**
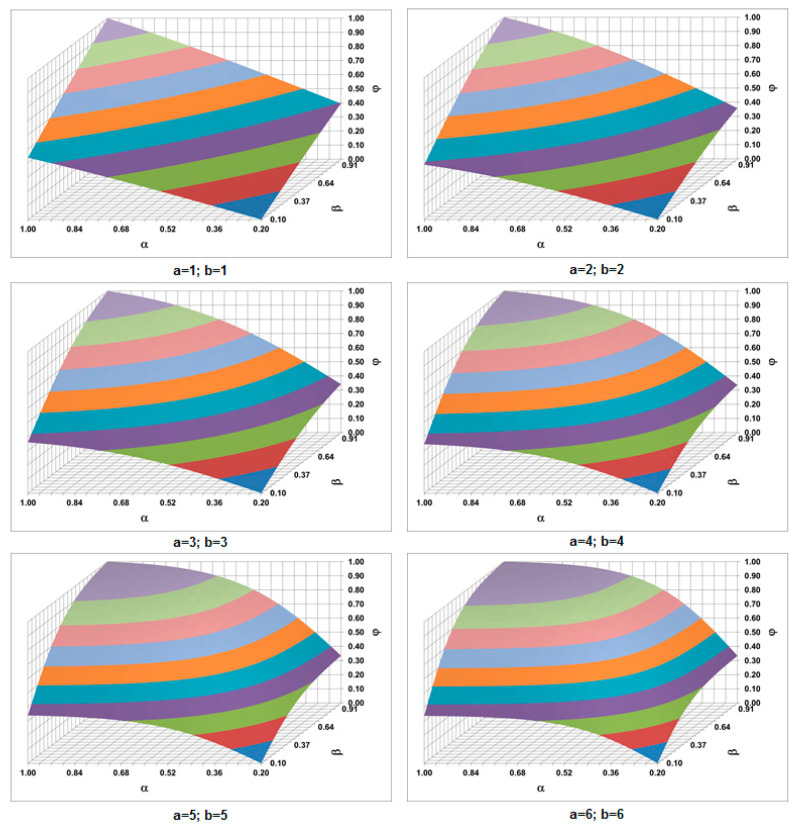
Different shapes of sigmoid-like function depending on a, b values (α_0_ = 0.2; β_0_ = 0.1).

Figure 4 shows the shape of the sigmoid-like function depending on the a and b parameters.

### 2.3. Callus Mesh Growth Algorithm

In this phase, the new nodes and elements corresponding to the evolution of callus growth in the corresponding simulation step will be generated. The generation of the new mesh elements is based on the initial positioning of their centroid, which will serve as the elemental reference point.

**Figure 5:**
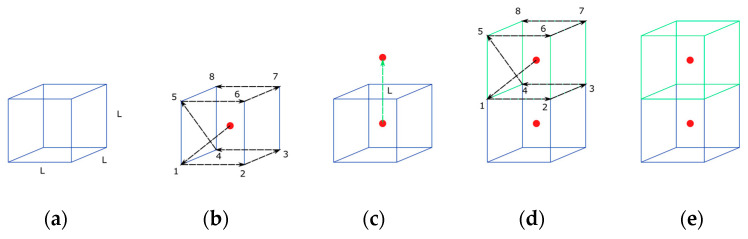
Connectivity and element generation for the 3D: (**a**) initial voxel element; (**b**) path vectors; (**c**) position of the new centroid; (**d**) new nodes and connectivity definition; (**e**) element attributes.

The first step is to calculate the path vectors, as shown in Figure 5b. The path vectors are the vectors that link one point to the next. They are calculated by taking the centroid as a first reference and proceeding to the next node of the element connectivity. Passing through all the nodes of the element, the nodes’ positions are settled for future element creation, taking each new centroid as a reference. This step is performed before the starting of the iteration through the growing elements. Once the path vectors have been calculated, the next step is the growth in the free directions. 

The free faces are calculated in the previous phase. As shown in Figure 5c, if there is no centroid in some direction and the element is allowed to grow, then a centroid is created with a distance L equal to the length of the side of the reference cube.

Once the centroid has been created, the new nodes and the connectivity are calculated. To do this, taking the centroid as the reference, the path vectors start to locate the points of the new element. If there is a node in that position, then the point is added to the connectivity of the element. If there is no node, then a node is created and added to the connectivity. The process is represented in Figure 5d.

Once the element is created, the material properties are assigned and the type of element is settled. This occurs in the last part of the element creation and corresponds to the event represented in Figure 5e. The geometric algorithm includes several checks in order to avoid element overlapping, both in the reference bone fragment and in the opposite bone fragment, controlling the position of new nodes. To ensure the appropriate connectivity of the mesh, it is verified that no nodes are repeated at the same position. If repeated nodes are detected, then the extra nodes are removed and a single node is used for the connectivity of all coincident elements at this node.

**Figure 6:**
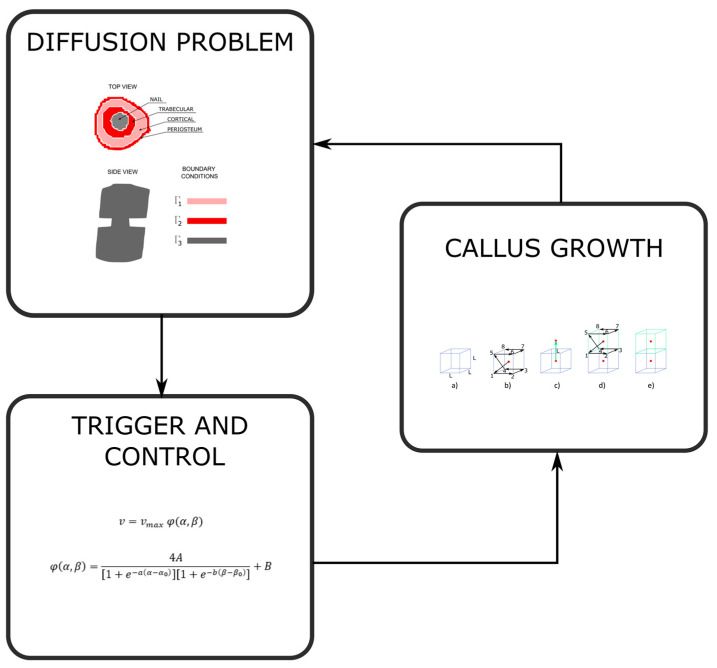
Explanatory diagram of the flux of the program for each time iteration.

The Python 3.8 language [40] was employed to control the workflow of the main program. As shown in Figure 6, the workflow for each time iteration has the three phases, as explained above in Section 2. The concentrations are calculated from the finite element model. In the trigger and control phase, the elements with sufficient concentration and with free faces are found. Finally, the callus growth takes place in those elements and the cycle restarts until the final iteration is achieved. Each one of these phases was programmed in FORTRAN [35].

## 3. Results

Several femoral types of fractures were chosen to apply the proposed approach to test the performance of the algorithm in the diaphyseal area treated through an IM nail (11 mm diameter). Three of the most common femoral fracture types were analysed, as explained in the following. The colours represent the cell concentrations. Navy blue is the lowest concentration and red the highest.

The values of the diffusion coefficient D related to the different parts of the bone are specified in Table 1 [16].

The values of the parameters of the sigmoid-like function are specified in Table 2 [16].

**Figure 7:**
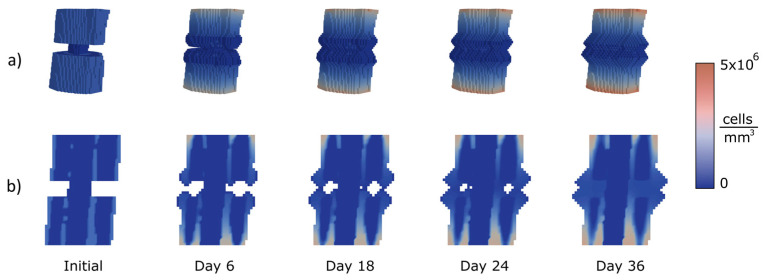
Calculation of concentrations (MSCs) and growth for a transverse fracture with 6 mm gap: (**a**) 3D view of the callus growth process; (**b**) sagittal section along femoral axis.

The first analysed case is a transverse fracture. A section of the femur with a length of 43 mm was considered. The initial gap is 6 mm high, and a voxel model was employed with a mesh size of 1 mm. Figure 7 shows the evolution of the callus growth in the transverse fracture. In the figure, the growth starts to be appreciable at day 6. At day 18, the link between the two broken parts begins. At that point, the callus surrounds the nail and the largest growth occurs near the periosteum, which coincides with the clinical evidence. At the end (day 36), the gap is closed, the cavity being filled seamlessly.

**Figure 8:**
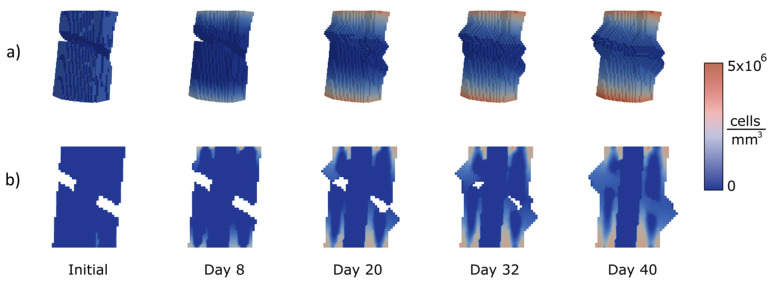
Calculation of concentrations (MSCs) and growth for an oblique fracture. 30° slope and 3 mm height: (**a**) 3D view of the callus growth process; (**b**) sagittal section along femoral axis.

The second case (Figure 8) corresponds to an oblique fracture with a 30° slope and a gap of 3 mm. The sequence of images shows the evolution of the callus over 40 days. As shown in the figure, the callus starts to grow near the lowest and the highest points of the facture. At day 32, the gap is almost filled but voids remain in its interior. Finally, at day 40 the gap is closed and the callus complete.

**Figure 9:**
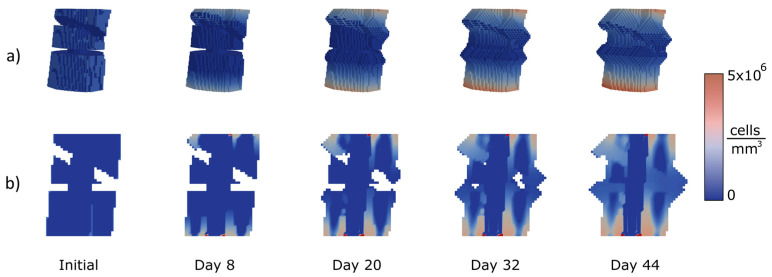
Calculation of concentrations (MSCs) and growth for a comminuted fracture with a combination of a 30° slope and a transverse shape with 3 mm gap: (**a**) 3D view of the callus growth process; (**b**) Sagittal section along femoral axis.

The third case is a comminuted fracture (Figure 9). This is a combination of a transverse fracture and an oblique fracture with a 30° slope and 3 mm gap. The images show how the callus grows until both sections of the bone and the comminuted fragment are connected. The comminuted fragment is inert and has no concentration nor production of cells. The gap between the upper boundary frontier, the comminuted fragment and the lower boundary starts to close in the active parts. The comminute is simply a bridge between parts that allows the fracture to close the gap, preventing non-union. At day 8, the highest point starts to grow the callus. At day 20, the growth of both frontiers can be appreciated. At day 32, the gap is almost closed, and at 44 days the comminuted fracture is totally closed. 

## 4. Discussion

A 3D FEA methodology was presented to simulate the initial phase of callus formation controlled by different biological parameters. For this purpose, a diffusion model was used to determine the cellular concentration in the fracture edges (bone and callus), which controlled the mesh growth algorithm, allowing the simulation of the progressive closure of the fracture gap. It should be noted that the model does not start from a pre-meshed domain, but rather the mesh grows freely by addition to the boundary of new elements. This 3D approach allows a better understanding of the problem, allowing more realistic simulations.

The main chosen cells were mesenchymal stem cells (MSC) and chondrocytes. Meanwhile, the molecules chosen to control the growth velocity were the TNF-α and the bone morphogenetic protein 2 (BMP-2). However, the model allows different biological magnitudes to be incorporated and adjusted according to the user’s needs.

The application cases described correspond to three of the most common femoral fracture types [41], stabilized using intramedullary nailing. 

Several works in the literature follow a different approach to bone healing simulation. The approach is applied in 2D and also in 3D models, and consists of pre-meshing the domain and changing the properties of the elements [15,17,19,21,22,23,24,25,26,27] according to fluid pressure, stress and strain. Some trials have been carried out to obtain more realistic shapes through this type of simulation. Schwarzenberg et al. [26] tried to obtain better results by applying a mechanoregulated model to this type of simulation. However, none of the above works include growing algorithms allowing free callus growth, therefore requiring pre-meshed domains.

It is difficult to compare the results obtained with those published by other authors, because none of the published approaches include free mesh growing. However, the final shape of the callus is very similar to the ones obtained with the diaphyseal simulation that appears in [7,24]. Concerning experimental results, in [26] results corresponding to an ovine tibia are presented, but they are not comparable with the results obtained for a human femur. Finally, the results obtained are in accordance with the radiographic images reported from fractures similar to the simulated ones.

The proposed approach to solve the growth problem over time avoids the use of external regulatory models, obtaining more natural results. The approach in this work gives a new understanding of the initial callus growth problem during fracture healing, and it has shown the potential to solve different fracture types and geometries. The algorithm is able to close the fracture gap even with isolated fragments, as in the case of comminuted fractures. 

The potential clinical value of this approach lies in helping orthopaedic surgeons to predict the times required for fracture callus formation depending on the type and complexity of the fracture, through personalized simulation of specific patients and fractures, for example from CT images. In this respect, 3D personalized surgery is evolving rapidly, orienting its efforts toward the patient [42], and the finite element method is gaining more weight in this research field [15,16,17,18,19,20,21,22,23,24,25,26,27,28,29,30,31,42,43].

The main limitation of the present study is that, for the moment, the implemented 3D approach is able to reproduce the generation of the primary callus, which corresponds to the initial phase of fracture healing, but not the complete fracture healing process. To complete the process, it would be necessary to incorporate the bone remodelling stage into the finite element model. For this purpose, a formulation that includes tissue differentiation and transformation should be added and implemented. This constitutes an objective for the improvement of the presented 3D approach to be developed in the forthcoming stages of the research, adding a complete bone remodelling model, including new phases in the global algorithm.

## 5. Conclusions

A complete 3D approach to simulate the initial callus growth during fracture healing in long bones has been presented. It could therefore be applied to the simulation of customized fractures for each individual patient. Thus, it would be possible to computationally predict the evolution of callus growth for the particular osteosynthesis of each specific case.

The proposed 3D approach has yielded reliable results in all the cases analysed, simulating the initial phase of callus generation previous to bone remodelling and consolidation. The model is able to completely close the fracture gap independently of the 3D geometry and fracture type for different biological conditions. Moreover, since callus growth is achieved by the addition of new elements to the fracture edge, instead of by activation (models with a pre-meshed domain), a more natural callus growth is obtained, without following pre-established geometric shapes. The proposed approach can be applied to the most complex bone fractures such as oblique, severely comminuted or spiral-type fractures, whose simulation remains hardly possible by means of the different existing approaches available to date.

## Figures and Tables

**Table 1:** Values of the diffusion coefficients inside the different regions of the bone. The values are constant throughout the corresponding regions.

PART	*D* Coefficient (µm^2^/min)
Bone marrow	100
Periosteum	100
Callus	50
Cortical bone	1

**Table 2:** Values of the parameters of the sigmoid-like function.

v_max_	5 mm^3^/day
a	5
b	5
*α*_0_	1/32
*β*_0_	1/16

## Data Availability

Not applicable.

## Abbreviations

IM	Intramedullary nail
MRI	Magnetic Resonance Imaging
MSCs	Mesenchymal stem cells
TNF-α	Tumour Necrosis Factor α
BMPs	Bone morphogenetic proteins
BMP-2	Bone Morphogenetic Protein 2
FEA	Finite Element Analysis
